# Barriers Composed of tRNA Genes Can Complement the Benefits of a Ubiquitous Chromatin Opening Element to Enhance Transgene Expression

**DOI:** 10.1002/biot.202400455

**Published:** 2025-02-16

**Authors:** Rebecca E. Sizer, Richard M. Ingram, Robert J. White

**Affiliations:** ^1^ Department of Biology University of York York UK

**Keywords:** barrier element, biomanufacturing, CHO cells, epigenetics, gene silencing

## Abstract

Random integration of transgenes into host cell genomes often occurs in epigenetically unstable regions, leading to variable and unreliable transgene expression. To address this, biomanufacturing organizations frequently employ barrier elements, such as the widely‐used ubiquitous chromatin opening element (UCOE). We have compared UCOE barrier activity against a barrier provided by tRNA genes. We demonstrate that the tRNA genes provide a more effective barrier than a UCOE in preventing transgene silencing in Chinese hamster ovary (CHO) cells. Nevertheless, the UCOE offers other benefits, increasing expression strongly, albeit transiently, and reducing production variability. Both the UCOE and tRNA genes counteract the repressive heterochromatin mark H3K9me3, but only the tRNA genes sustain euchromatic H3K27ac and recruitment of RNA polymerase II (Pol II) throughout long‐term culture. A hybrid combining these distinct types of elements can provide benefits of both, enhancing expression in a more enduring manner. This synthetic hybrid offers potential for biomanufacturing applications.

## Graphical Abstract and Lay Summary

A major challenge for the biopharmaceutical industry is instability of transgene expression, where epigenetic repression silences genes encoding high‐value products. We demonstrate that specific tRNA genes (tDNAs) can mitigate this widespread problem in chinese hamster ovary (CHO) cells, excluding the repressive epigenetic modification H3K9me3, and promoting the addition of the activating modification H3K27ac. When tested in parallel, a ubiquitous chromatin opening element (UCOE) also excludes H3K9me3, but is less effective than the tDNAs at promoting H3K27ac addition.  

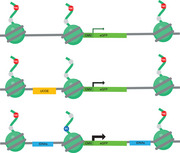

## 1 Introduction

A significant proportion of biopharmaceutical products are manufactured recombinantly in mammalian cells, primarily Chinese hamster ovary (CHO) cells [1]. While CHO cells present numerous advantages, including high productivity, capacity for suitable post‐translational modifications, and low susceptibility to human viruses, their inherent production instability poses a significant challenge.

Production instability results in unpredictable decreases in expression over prolonged culture. It is frequently caused by epigenetic silencing, the non‐mutational inactivation of genes through repressive covalent modifications, including DNA methylation and histone deacetylation. [2, 3, 4, 5, 6, 7, 8, 9, 10, 11] These modifications can act independently or in combination to determine transcriptional activity [12, 13, 14, 15]. Aberrant DNA methylation and hypoacetylation of histone H3 at recombinant transgenes have been implicated in variegated transgene expression, which can lead to complete silencing over time [4, 6, 7, 10, 16, 17].

Production instability caused by epigenetic silencing can be ameliorated by genetic elements termed barriers. These fall into two main categories: (i) those that impede the spread of heterochromatin into euchromatic regions (e.g., stabilizing anti‐repressor elements [STAR] and scaffold or matrix attachment regions [S/MAR) elements]), and (ii) those that actively modify chromatin to enhance transcriptional activity, (e.g., ubiquitous chromatin opening elements [UCOEs]). A comparative study demonstrated that a UCOE surpassed other commonly used barriers at enhancing and stabilizing transgene expression [18].

UCOEs were originally identified at the ubiquitously expressed human TBP‐PSMB1 and HNRPA2B1‐CBX3 (A2UCOE) loci, but have since been identified at further sites, such as the mouse Rps3 locus [19, 20, 21, 22, 23, 24]. They are widely used in protein biomanufacturing, facilitating generation of high‐expressing recombinant cell clones. The A2UCOE and the Rps3 UCOE have demonstrated effectiveness in enhancing expression stability over extended culture periods across various industrially‐relevant cell lines, including different CHO cell lineages [25, 26, 27, 28]. Despite their success, the effectiveness of UCOEs is context‐dependent [29, 30].

A pair of tRNA gene clusters, referred to as the ALOXE3 and TMEM107 (AT) clusters, was recently shown to enhance transgene expression stability upon random integration into CHO cells [31]. These clusters elevated cell‐specific productivity by up to 3‐fold and augmented the number of stably‐expressing cells by approximately 30%, raising the titer of monoclonal antibodies and an Fc‐fusion [31]. They also exhibit barrier properties in human cells and *Schizosaccharomyces pombe*, demonstrating functionality across diverse contexts [32].

This study evaluates the efficacy of novel tRNA gene barriers in protecting a transgene from silencing compared to the industrially utilized UCOE. We find that the AT tRNA gene clusters surpass the UCOE as a barrier, whereas the UCOE is beneficial in transiently increasing median transgene expression. Mechanistic investigations revealed that both the AT and UCOE barriers enhance promoter accessibility, prevent constitutive heterochromatic silencing, and boost the euchromatic mark H3K27ac at a linked transgene. However, only the AT sustains the increase in H3K27ac over long‐term culture. A combination of these elements can enhance and sustain transgene expression.

## 2 Materials and Methods

### 2.1 Cloning and Linearization

**FIGURE 1:**
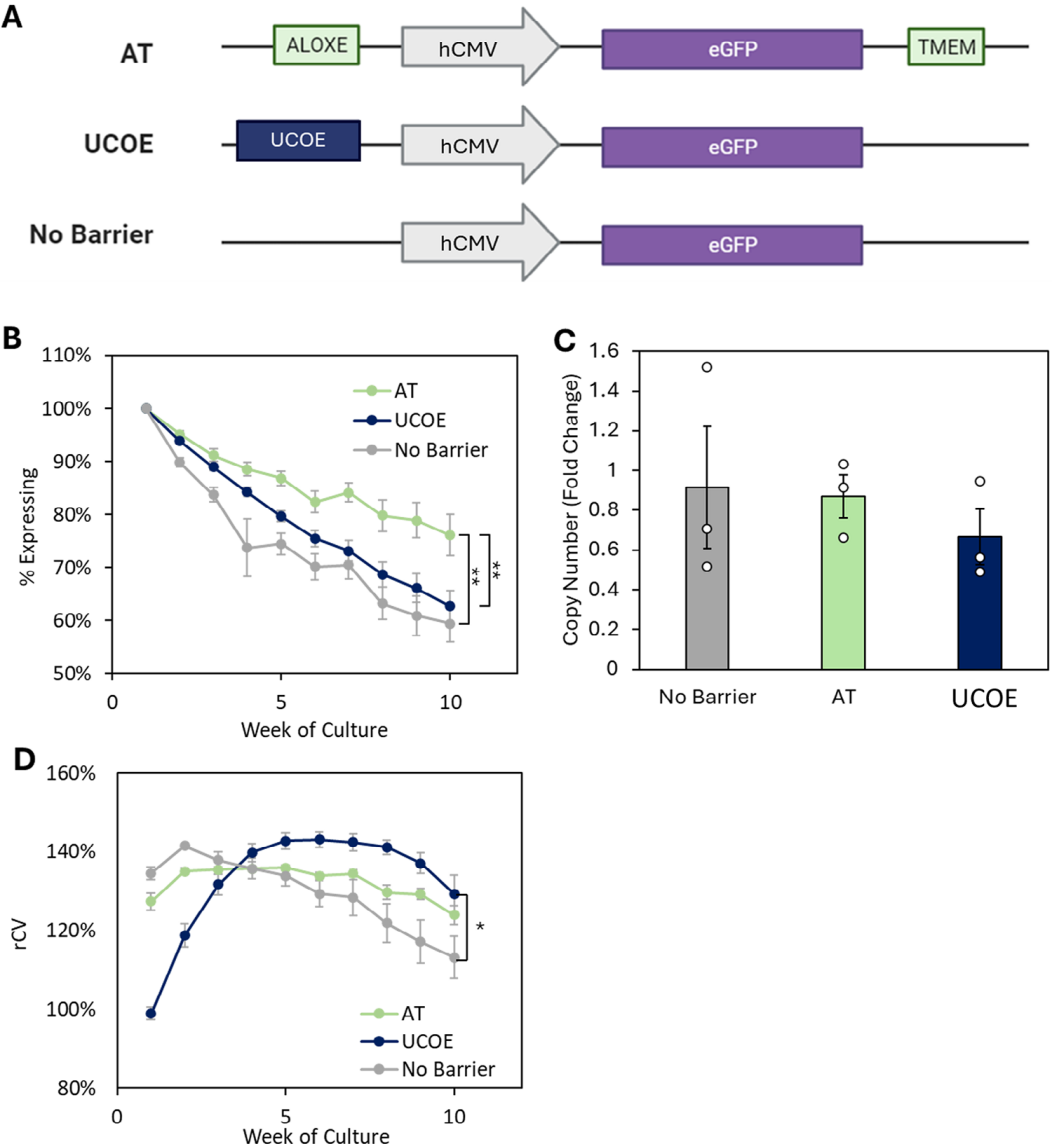
tRNA gene barriers increase transgene expression stability. CHO‐K1 cells were transfected with vectors containing an eGFP reporter gene driven by the human CMV promoter and flanked as illustrated in (A). Stable transfectants were selected for 2 weeks and then eGFP expression was monitored from cell pools for 10 weeks by flow cytometry. (A) Schematic representation of constructs. (B) Percentages of cells positive for eGFP. (C) qPCR analysis of eGFP copy number at early and late time points. *n* = 3. (D) Time course of rCV values for each construct. * denotes *p* < 0.05, ** denotes *p* < 0.01 (*t‐*test). Error bars = SEM.

The Rps3 UCOE expression vector (mouse 3.2 kb Puro Set) was purchased from Merck (504865, Sigma‐Aldrich). Details of the tRNA gene fragments are described by Sizer et al. [31]. UCOE and tRNA gene fragments were inserted into a backbone vector with an hCMV‐driven eGFP gene in the configurations shown in Figures 1A and 5A. Plasmids were linearized prior to transfection by digestion with a restriction enzyme (PvuI or NotI) that recognizes a unique site.

### 2.2 Cell Culture

CHO‐K1 cells were cultured as described previously in [31]. Transfection utilized Xfect Transfection reagent (631318, TakaRa) following the manufacturer's instructions. Briefly, cells were seeded at a density of 3 × 10^6^ in a 10 cm^2^ six‐well plate 24 h before transfection. Each plasmid (5 µg) was mixed with Xfect Reaction Buffer and polymer to a total volume of 100 µL per transfection. The nanoparticle mixture (100 µL) was added dropwise to the cells. After 48 h, cells were transferred to a T75 flask with 15 mL of media containing 10 µg/mL puromycin (A11138‐03, Gibco) for selection of stably transfected cells. Selection pressure was maintained for 2 weeks post‐transfection.

### 2.3 Flow Cytometry

Flow cytometry analysis of eGFP was conducted every 7 days over 10 weeks using a CytoFLEX LX flow cytometer. Analysis involved resuspending 500,000 cells in 500 µL of media for each sample. Unstained cells were selected based on their forward and side scatter characteristics to isolate the desired population. Acquisition and stopping settings were configured to achieve a cell count of 20,000, with adjustments where necessary. Consistent settings were maintained throughout the experiment. Subsequent analysis was performed using CytExpert software (version 2.4.0.28).

### 2.4 Copy Number Quantification

Transgene copy number was determined by quantitative polymerase chain reaction (qPCR) relative to a plasmid standard curve, as described in [31].

### 2.5 RNA Extraction and cDNA Synthesis

RNA extraction and cDNA synthesis were carried out as described previously in [31].

### 2.6 Chromatin Immunoprecipitation

The chromatin immunoprecipitation method was modified from Ghisletti et al. [33]. Cells were grown to approximately 90% confluence in T175 flasks and crosslinked with 1% formaldehyde. Crosslinking was stopped with TBS/glycine/EDTA solution, followed by cell lysis. Chromatin was sheared by sonication and debris was removed by centrifugation. The chromatin was then precleared and immunoprecipitated with specific antibodies coupled to G Dynase beads (10004D, Invitrogen). After overnight incubation, the beads were washed and DNA eluted. Crosslinking was reversed overnight and DNA was purified using SPRI beads (A63881) and quantified using qPCR. Specific antibody details are listed in Table 1.

**TABLE 1:** Antibodies used for ChIP.

Target	Manufacturer	Reference
H3	Abcam	AB1791
H3K27ac	Abcam	AB4729
H3K27me3	Abcam	AB6002
H3K9ac	Abcam	AB4441
H3K9me3	Abcam	AB8898
Pol II	Abcam	ab817

### 2.7 Formaldehyde Assisted Isolation of Regulatory Elements (FAIRE)

After chromatin had been extracted as above for chromatin immunoprecipitation, FAIRE was conducted according to the method outlined by Simon et al. [34]. Chromatin from ∼1 × 10⁷ cells was subjected to a phenol/chloroform extraction to separate high protein‐bound DNA from low protein‐bound DNA. The aqueous phase containing the low protein‐bound DNA was then precipitated by adding 1/10 volume of 3 M sodium acetate and two volumes of ethanol, followed by incubation at −80°C for at least 1 h. The precipitated DNA was resuspended in 10 mM Tris‐HCl (pH 7.4).

Subsequently, 1 µL of DNase free RNase and proteinase K were added to the sample to degrade any residual proteins and mRNA. Crosslinking was reversed by incubating the samples overnight at 56°C. DNA purification was performed using SPRI beads according to the manufacturer's instructions, with elution in water. After reverse crosslinking and an additional phenol‐chloroform extraction to ensure sample purity, the purified FAIRE‐DNA was quantified by qPCR using CMV primers (Table 2).

**TABLE 2:** List of primers used for ChIP, FAIRE, RNA, and copy number analysis.

Target	Direction	Sequence
eGFP	Forward	AGTCCGCCCTGAGCAAAGA
	Reverse	TCCAGCAGGACCATGTGATC
CMV	Forward	ATGTCGTAACAACTCCGCCC
	Reverse	TAGCGGATCTGACGGTTCAC
5.8S rRNA	Forward	TCGATGAACGCAGCTA
	Reverse	GTGCGTTCGAAGTGTCGA
GapDH	Forward	TTCTAGAGACAGCCGCATC
	Reverse	CCCCGTTTCCGACCGT

### 2.8 qPCR

Details of qPCR were as described in [31]. Primers are detailed in Table 2.

## 3 Results

### 3.1 tRNA Gene Barriers Outperform a UCOE at Sustaining Transgene Expression

Barrier properties of the AT tRNA genes were compared with those of a UCOE using an eGFP reporter assay. Vectors contained an eGFP gene either (i) flanked by AT tRNA gene barrier clusters, (ii) placed downstream of a 3.2 kb UCOE fragment, or (iii) without barrier elements (Figure 1A). A single upstream UCOE was used because this is recommended by its manufacturer, although other arrangements can also be beneficial [18]. Integration of exogenous DNA into the CHO genome generally occurs in tandem arrays, with the result that most transgenes will become flanked by UCOEs, the downstream element provided by an adjacent repeat in the array. The constructs were linearized and transfected into CHO‐K1 cells. Stable transfectants were selected using puromycin resistance, and eGFP expression was monitored by flow cytometry for 10 weeks after withdrawal of selection. The AT barrier mitigated the loss of eGFP‐expressing cells, maintaining 20% more eGFP+ cells at Week 10 than the control (Figure 1B). In contrast, the UCOE showed limited efficacy, only maintaining expression in 5% more cells than the control, significantly less than those with the AT tRNA genes (Figure 1B). These differences cannot be attributed to variations in the loss of eGFP transgene copies (Figure 1C).

Heterogeneity within each population was determined as the robust coefficient of variation (rCV), which is calculated as the 75th percentile minus the 25th percentile, divided by the median. In the absence of a barrier, the rCV progressively declined during prolonged culture (Figure 1D). This likely reflects a loss of high‐expressing cells from the population. In contrast, the AT element maintained a higher rCV. With the UCOE, the rCV was initially lower, indicating less variability in eGFP expression, but it gradually increased significantly, possibly reflecting emergence of cell populations that did not express eGFP.

**FIGURE 2:**
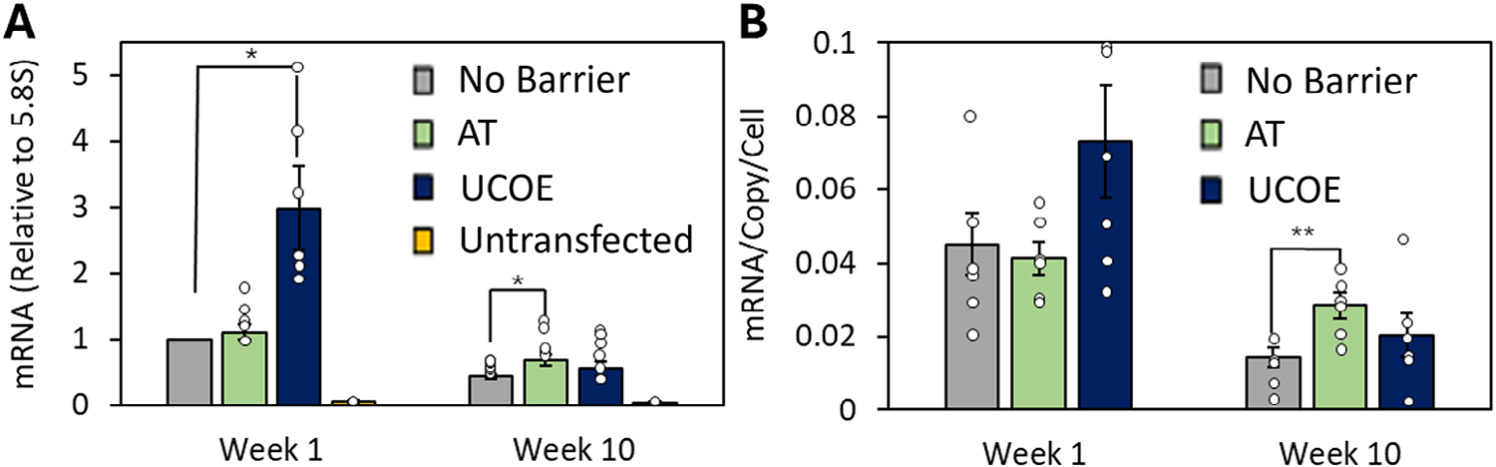
tRNA gene barriers maintain transgene mRNA expression during prolonged culture. CHO‐K1 cells were transfected with vectors encoding an eGFP reporter gene driven by a CMV promoter and flanked by the AT tRNA gene barriers, placed downstream of the UCOE, or devoid of any barrier elements. Stable cells were selected for 2 weeks, and eGFP expression was analyzed at an early (Week 1) and late (Week 10) time point. (A) RT‐qPCR analysis of eGFP mRNA expression at an early and late time point normalized to 5.8S rRNA. (B) Bar chart showing amount of eGFP mRNA produced per cell per copy of the eGFP transgene at early and late time points. *n* = 6, * denotes *p* < 0.05, ** denotes *p* < 0.01 (*t*‐test). Error bars = SEM.

RT‐qPCR was used to quantify transgene mRNA levels at early (Week 1) and late (Week 10) time points. The UCOE significantly increased transgene mRNA levels, ∼3‐fold over control levels at Week 1, but this increase was lost over long‐term culture (Figure 2A). In contrast, the AT barrier did not affect eGFP mRNA levels at Week 1, but by Week 10, cells containing the AT barrier expressed eGFP 1.5‐fold more strongly than the control cells. This corresponds to 2‐fold higher expression per gene when normalized to copy number (Figure 2B).

In keeping with these mRNA data, flow cytometry demonstrated that the UCOE boosted eGFP fluorescence in Week 1, relative to the no barrier and AT constructs (Figure S1). However, large numbers of UCOE cells had minimal fluorescence by Week 10, as with the no barrier population. In contrast, the AT construct allowed most cells to maintain fluorescence at Week 10.

These results demonstrate robust barrier properties of the AT construct, effectively mitigating the loss of transgene expression otherwise observed over long‐term culture. In contrast, the UCOE displayed much weaker barrier activity, but significantly enhanced expression and reduced early variation in expression.

### 3.2 Barriers Maintain an Open Chromatin Configuration

**FIGURE 3:**
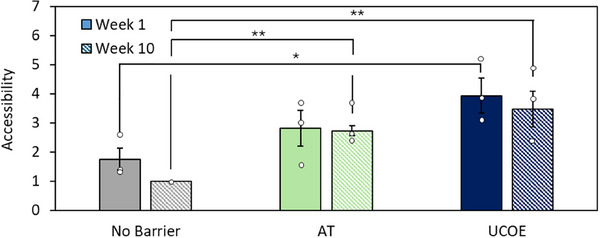
Barriers maintain promoter accessibility over long term culture. CHO‐K1 cells were transfected with vectors containing an eGFP reporter gene driven by a CMV promoter and flanked by the AT tRNA gene barriers, placed downstream of the UCOE, or without any barrier. Stable cells were selected and then promoter accessibility was determined by FAIRE‐seq one and 10 weeks after selection was removed. Numerical values are ratios of accessibility relative to the no barrier control after 10 weeks without selection. *n* = 3–4, * denotes *p* < 0.05, ** denotes *p* < 0.01 (*t‐*test). Error bars = SEM.

Increased transcription commonly correlates with increased chromatin accessibility [34]. FAIRE‐qPCR is a technique used to identify regions of open chromatin by isolating and quantifying DNA sequences that are relatively accessible because they are not bound by nucleosomes [35]. This revealed that in the absence of a barrier, promoter accessibility decreased over long‐term culture, correlating with the fall in eGFP mRNA (Figure 3). In contrast, inclusion of the AT or UCOE barriers led to a 2.7‐fold and 3.4‐fold increase in promoter accessibility, respectively, compared to the control at Week 10. The UCOE also significantly enhanced promoter accessibility at Week 1, relative to when no barrier was included, aligning with the increase in mRNA levels at this stage (Figure 3).

### 3.3 AT tRNA Gene Barriers and UCOE Modify the Epigenetic Environment Differently

**FIGURE 4:**
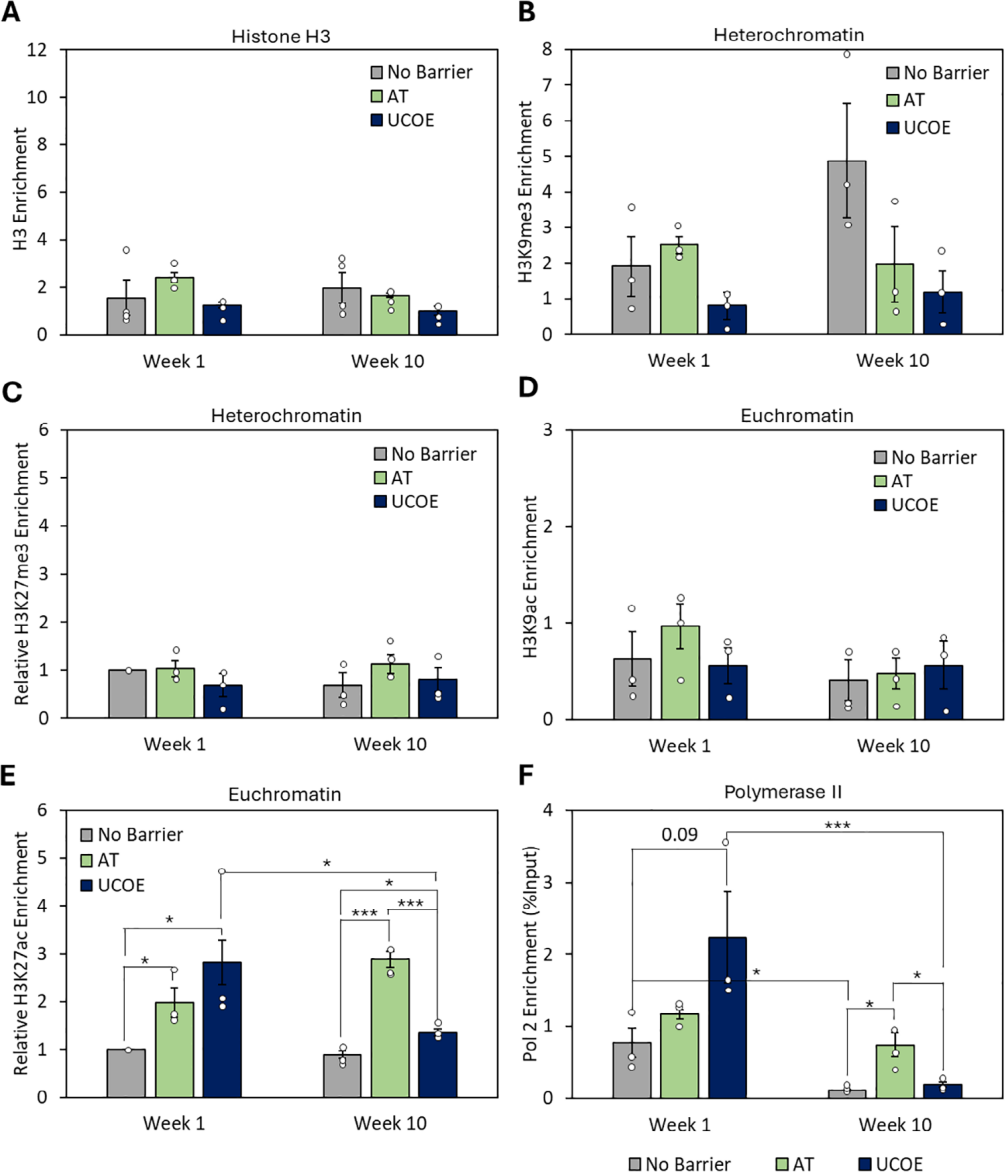
Inclusion of barriers alters the epigenetic environment of transgenes. Cells were transfected with vectors containing an eGFP reporter gene driven by a CMV promoter and with the AT tRNA genes, UCOE, or without any barrier. Stable cells were selected for 2 weeks and then histone modifications at the transgene were analyzed at week 1 and week 10. Bar chart showing ChIP‐qPCR results for H3 (A), H3K9me3 (B), H3K27me3 (C), H3K9ac (D), H3K27ac (E) at the eGFP transgene normalized to input and H3. (F) Bar chart showing ChIP‐qPCR results for RNA polymerase II at the hCMV promoter, normalized to input. *n* = 3–4, * denotes *p* < 0.05, ** denotes *p* < 0.01, *** denotes *p* < 0.001. Error bars = SEM.

Multiple studies have linked production instability to epigenetic silencing [4, 6, 7, 10, 16, 17]. Indeed, methylation of histone H3 at lysine 9 (H3K9me3) or 27 (H3K27me3) correlates strongly with loss of DNA accessibility and transcription [36, 37]. These heterochromatic modifications were measured at the transgene promoter by ChIP‐qPCR, along with the level of histone H3. While H3 and H3K27me3 changed little, there was a 2.6‐fold increase in H3K9me3 over long‐term culture when no barrier was present (Figure 4A–C). This suggests that H3K9me3‐based heterochromatin contributes to transgene silencing in this context, rather than facultative H3K27me3‐based heterochromatin. Both the AT and UCOE barriers prevented the increase in H3K9me3 during culture (Figure 4B). That mRNA levels still fall when the UCOE blocks H3K9me3 acquisition suggests that heterochromatinization is not required for the decreased transgene expression during prolonged culture.

Acetylation of lysine 9 or 27 of histone H3 can boost gene expression directly [38, 39]. Although minimal changes were observed for H3K9ac, both the AT and UCOE significantly enhanced H3K27ac enrichment at the transgene, showing 2‐fold and 3‐fold increases over control at Week 1, respectively (Figure 4D–E). The AT barrier maintained this increase over 10 weeks, consistent with the sustained number of GFP+ cells and increased mRNA. In contrast, the UCOE did not prevent a significant fall in H3K27ac (Figure 4E).

Together, these results show that both the AT tRNA genes and UCOE can block H3K9me3‐mediated heterochromatic silencing, but this does not prevent transgene repression over long‐term culture. Maintenance of transgene expression correlates with persistence of H3K27ac, a modification that boosts transcription. This supports previous evidence that high H3K27ac correlates with resistance to long‐term silencing. [8, 40]

### 3.4 Barrier Elements Boost Pol II Occupancy at Transgenes

ChIP‐qPCR was used to investigate the effects of barriers on recruitment of RNA polymerase II (Pol II), which is responsible for transcribing protein‐coding genes. In the absence of a barrier, there was a significant decrease in Pol II at the transgene during culture, consistent with the fall in its mRNA (Figure 4F). However, the AT barrier mitigated this decline, retaining Pol II at the transgene. In line with the mRNA data in Figure 2, the UCOE initially boosted Pol II binding over control levels, but this increase disappeared during long‐term culture. Thus, the AT tRNA genes sustain Pol II at the transgene over long‐term culture, consistent with their barrier function, whereas the UCOE enhances recruitment initially, but its effect diminishes over prolonged culture.

### 3.5 Barrier Elements can be Combined to Create Optimized Vectors

**FIGURE 5:**
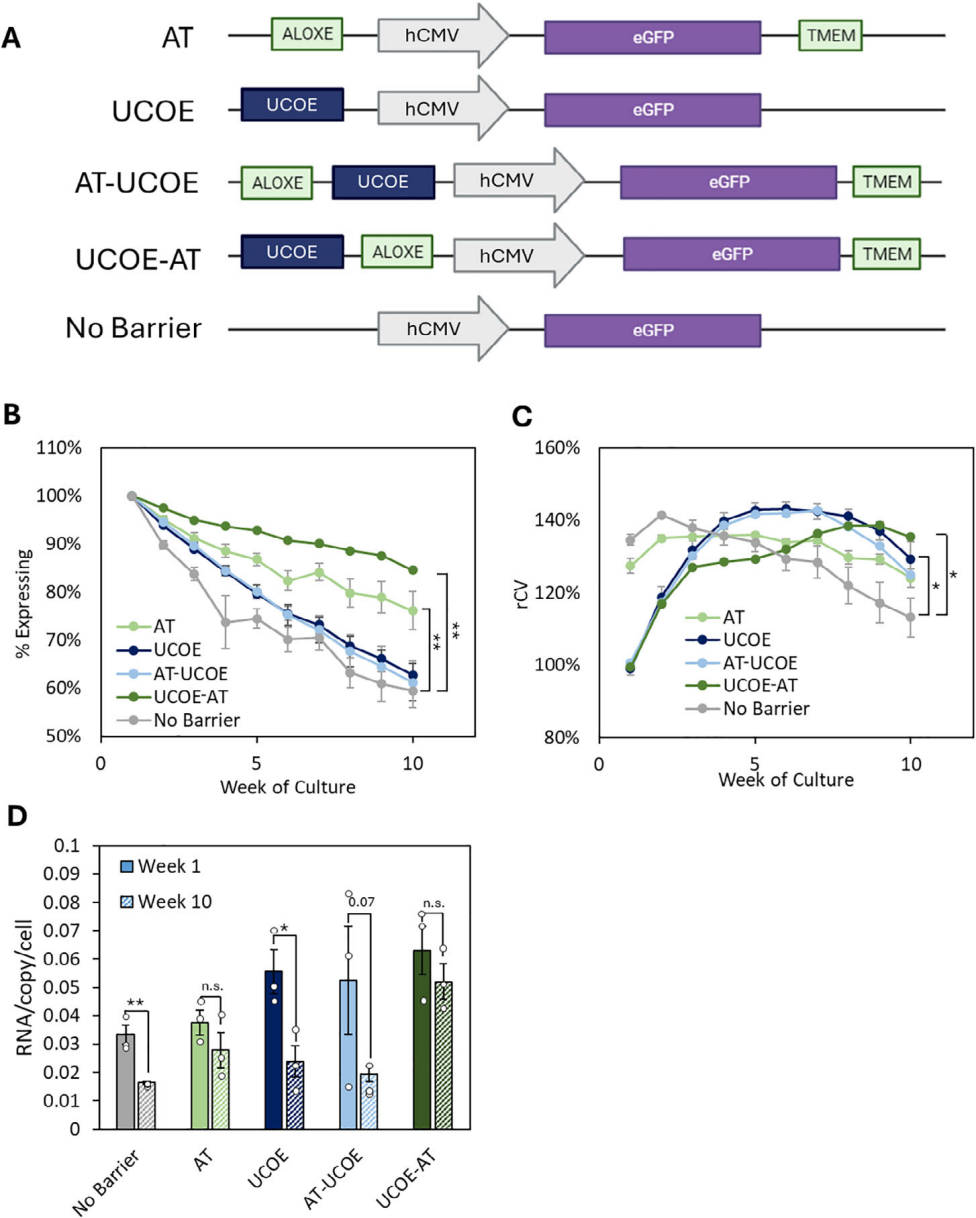
Genetic elements can be combined to enhance and sustain expression. Cells were transfected with vectors containing an eGFP reporter gene driven by the human CMV promoter and with the AT tRNA genes and/or UCOE, in the relative positions indicated, or without any barrier. Stable transfectants were selected for 2 weeks and then eGFP expression was monitored by flow cytometry for 10 weeks after removal from selection. (A) Schematic showing constructs used. (B–C) Line graphs showing the percentage of cells that were positive for eGFP (B) and the robust coefficient of variation (C) over long‐term culture. (D) RT‐qPCR results quantifying eGFP mRNA at early and late time points, normalized to 5.8 rRNA and the number of transgene copies per cell. *n* = 3, * denotes *p* < 0.05, ** denotes *p* < 0.01 (*t*‐test). Error bars = SEM.

As the AT and UCOE barriers offer distinct benefits for transgene expression, we explored potential synergies between them. We generated two synthetic barriers combining the AT tRNA gene clusters and UCOE in alternative configurations, with the ALOXE cluster of tRNA genes either upstream or downstream of the UCOE (Figure 5A). The UCOE‐AT construct, where the ALOXE cluster was placed downstream of the UCOE, increased the number of expressing cells at 10 weeks compared to either element alone and maintained expression in 25% more cells than the control lacking any barrier (Figure 5B). However, this enhancement was configuration‐dependent, as the AT‐UCOE construct gave no greater protection against transgene silencing than the UCOE alone. Both the AT‐UCOE and UCOE‐AT reduce the rCV at the start of long‐term culture, to a similar extent to the UCOE by itself (Figure 5C). RT‐qPCR showed that the AT‐UCOE and UCOE‐AT both boosted mRNA levels to a similar extent to the UCOE alone at the start of culture (Figure 5D). However, while expression fell substantially with the UCOE and AT‐UCOE constructs, similarly to the drop seen in the absence of a barrier, mRNA from the UCOE‐AT construct showed much less decrease and remained elevated after 10 weeks in culture (Figure 5D).

The data demonstrate favorable properties of UCOE‐AT, which combines the barrier activities of tRNA gene clusters with transcriptional enhancement by the UCOE, allowing sustained and elevated expression over long‐term culture.

## 4 Discussion

This study compares the properties of the AT tRNA gene barrier with those of a UCOE that is widely used in industry. It demonstrates the potential of highly conserved tRNA genes as effective barriers against epigenetic silencing. Under the conditions tested, the AT tRNA genes mitigate the fall in the number of transgene‐expressing cells more effectively during prolonged culture than the UCOE (Figure 1B). At the molecular level, the AT barrier sustained transgene mRNA expression and Pol II occupancy, functions not displayed by the UCOE despite an initial strong increase in mRNA level (Figures 2 and 4F). Sustained expression is preferable for biomanufacturing, rather than an early stronger but transient peak, as prolonged time in bioreactors can compromise product quality [41], a problem exasperated if production is highest earlier in the incubation. The greater ability of the AT tRNA gene barrier to maintain transgene expression could be especially beneficial where perfusion is used to prolong production runs and in high volume bioreactors, where the growth phase after seeding is longer.

Distinct benefits observed for the UCOE are a powerful early increase in transgene expression, albeit short‐term, and an initial reduction in expression variability (Figures 1D and 2). The reduced variability may reflect, at least in part, expression in cells where the transgenes might otherwise be silent. The presence of non‐expressing cells in a population can be extremely problematic, as they often grow faster than high producers [42]. Although some studies have found that UCOEs can improve expression stability, their barrier activity is sensitive to context [21, 28–30, 43–46] and was not evident under our assay conditions. As the transcription‐boosting effect occurs under conditions where its barrier activity is not apparent, the UCOE may have dual functions that can operate independently of each other, depending on conditions.

Both the UCOE and the AT tRNA gene barrier increase promoter accessibility and induce changes in epigenetic marks (Figures 3 and 4). This is consistent with the established relationship between DNA accessibility and some chromatin modifications [47]. The heterochromatin mark H3K9me3 is associated with inaccessibility and accumulated at unprotected transgenes in our assays, but this was mitigated by the tRNA genes or UCOE (Figure 4B). It is noteworthy that expression of transgenes linked to the UCOE was no longer enhanced after 10 weeks in culture, despite the continued suppression of H3K9me3 and maintenance of accessibility (Figures 2, 3, and 4B). However, the UCOE did not maintain the euchromatic mark H3K27ac, despite stimulating this strongly at Week 1 (Figure 4E). In contrast, the AT tRNA genes sustained H3K27ac and continued to stimulate Pol II recruitment after 10 weeks in culture, which may explain their superior performance at this late time point (Figure 4E,F). This important quality reflects the stable occupancy of tRNA genes by p300 and CBP, the histone acetyltransferases responsible for acetylating H3K27 [48, 49]. Acetylated histones provide binding sites for transcription factors with bromodomains, such as TFIID, which facilitate assembly on promoters of complexes that recruit Pol II, consistent with the significant enrichment of Pol II observed with the AT tRNA genes (Figure 4F).

We combined the AT tRNA gene barrier with the UCOE to create a hybrid, UCOE‐AT, which achieved 25% more GFP+ cells than the control, after 10 weeks of culture (Figure 5B). It also sustained a ∼2‐fold increase in expression at this late stage (Figure 5D). This hybrid therefore combined the key and distinct advantages of its component parts, the enhanced transcription obtained with a UCOE and the resistance to repression characteristic of the AT tRNA gene clusters. These beneficial effects depend on configuration, and barrier activity is compromised if the UCOE is positioned between the ALOXE3 tRNA gene cluster and the transgene, as in the AT‐UCOE hybrid (Figure 5B). It is unclear why this arrangement is unsuccessful, but it could be that the ALOXE3 barrier functions in a distance‐dependent manner. Alternatively, the UCOE may interfere with the barrier activity of the ALOXE3 cluster in this configuration. Integrated transgenes generally form tandem arrays, and interactions between elements from adjacent repeats may potentially impact efficacy. For example, if UCOE‐AT integrates as a head‐to‐tail array, transgenes could be influenced by the UCOE from the downstream repeat in addition to the UCOE placed upstream in the vector. Previous studies found that UCOE efficiency can be affected by its position and context [18, 50, 51]. For example, a UCOE was found to boost expression more strongly when located upstream of a transgene than when it was positioned downstream [18]. Another study found that a UCOE upstream of the CMV promoter stimulated mRNA expression more strongly from a transgene encoding an antibody light chain when compared to a transgene encoding an antibody heavy chain [51]. Nevertheless, both the AT‐UCOE and UCOE‐AT increase mRNA levels and reduce the rCV at an early time point, showing that these advantageous attributes of the UCOE are able to tolerate its different locations.

The epigenetic environment encountered by transgenes will vary according to their sites of integration. In this study, we opted to assay heterogeneous populations of transfected cells to test performance in a range of contexts. The effects reported here, therefore, represent averaged activity of transgenes integrated into a variety of sites. The possibility remains that individual clones with uniform integration sites may, in some cases, behave differently from the average behavior, due to atypical chromosomal conditions. We showed previously that the AT tRNA gene barriers perform well in the context of clonal cell lines, the standard conditions used in biomanufacturing [31]. Before UCOE‐AT hybrids can be exploited commercially, they will also need to be tested extensively under conditions more closely applicable to industrial use. The promising results reported here suggest that these further tests are warranted.

## 5 Conclusion

Our results demonstrate that the AT tRNA gene clusters provide a robust barrier that can protect against transgene silencing more effectively than a UCOE that is currently used industrially. Nevertheless, the UCOE provides clear benefits, increasing production initially and reducing the heterogeneity of expression between individual cells. Both elements increase the accessibility of chromatin and alter epigenetic marks to favor expression, suppressing the accumulation of H3K9me3, a feature of heterochromatin that mediates transcriptional repression. Our results also show that the UCOE can increase Pol II recruitment to the transgene, while the AT tRNA genes can minimize its loss over long‐term culture. When configured appropriately, a hybrid barrier combining the AT tRNA genes and the UCOE displays benefits from both elements, increasing and sustaining expression. This synthetic barrier holds potential for significantly enhancing and maintaining transgene expression in biomanufacturing applications.

## Author Contributions

**Rebecca E. Sizer**: conceptualization, data curation, formal analysis, investigation, methodology, validation, project administration, visualization, writing–original draft, writing–review and editing. **Richard M. Ingram**: methodology. **Robert J. White**: conceptualization, writing–review and editing, supervision, funding acquisition.

## Conflicts of Interest

The authors declare no conflicts of interest.

## Supporting information



Supporting Information

## Data Availability

The data that support the findings of this study are available from the corresponding author upon reasonable request.

## References

[1] G. Walsh and E. Walsh , “Biopharmaceutical Benchmarks 2022,” Nature Biotechnology 40, no. 12 (2022): 1722–1760. 10.1038/s41587-022-01582-x PMC9735008 36471135

[2] L. M. Barnes , C. M. Bentley , and A. J. Dickson , “Stability of Protein Production from Recombinant Mammalian Cells,” Biotechnology and Bioengineering 81, no. 6 (2003): 631–639. 12529877 10.1002/bit.10517

[3] C. H. Fann , F. Guirgis , G. Chen , M. S. Lao , and J. M. Piret , “Limitations to the Amplification and Stability of Human Tissue‐Type Plasminogen Activator Expression by Chinese Hamster Ovary Cells,” Biotechnology and Bioengineering 69, no. 2 (2000): 204–212. 10861399 10.1002/(sici)1097-0290(20000720)69:2<204::aid-bit9>3.0.co;2-z

[4] M. Kim , P. M. O'Callaghan , K. A. Droms , and D. C. James , “A Mechanistic Understanding of Production Instability in CHO Cell Lines Expressing Recombinant Monoclonal Antibodies,” Biotechnology and Bioengineering 108, no. 10 (2011): 2434–2446. 21538334 10.1002/bit.23189

[5] J. C. Eissenberg , “Position Effect Variegation in Drosophila: Towards a Genetics of Chromatin Assembly,” BioEssays: News and Reviews in Molecular, Cellular and Developmental Biology 11, no. 1 (1989): 14–17. 2505764 10.1002/bies.950110105

[6] Y. Yang , C. Mariati , and M. G. S. Yap , “DNA Methylation Contributes to Loss in Productivity of Monoclonal Antibody‐Producing CHO Cell Lines,” Journal of Biotechnology 147, no. 3–4 (2010): 180–185. 20430058 10.1016/j.jbiotec.2010.04.004

[7] N. Veith , H. Ziehr , R. A. F. MacLeod , and S. M. Reamon‐Buettner , “Mechanisms Underlying Epigenetic and Transcriptional Heterogeneity in Chinese Hamster Ovary (CHO) Cell Lines,” BMC Biotechnology 16 (2016): 6. 26800878 10.1186/s12896-016-0238-0 PMC4722726

[8] B. Moritz , L. Woltering , P. B. Becker , and U. Göpfert , “High Levels of Histone H3 Acetylation at the CMV Promoter Are Predictive of Stable Expression in Chinese Hamster Ovary Cells,” Biotechnology Progress 32, no. 3 (2016): 776–786. 27073121 10.1002/btpr.2271

[9] S. Spencer , A. Gugliotta , J. Koenitzer , H. Hauser , and D. Wirth , “Stability of Single Copy Transgene Expression in CHOK1 Cells Is Affected by Histone Modifications but Not by DNA Methylation,” Journal of Biotechnology 195 (2015): 15–29. 25533398 10.1016/j.jbiotec.2014.12.009

[10] J. Chusainow , Y. S. Yang , J. H. M. Yeo , et al., “A Study of Monoclonal Antibody‐Producing CHO Cell Lines: What Makes a Stable High Producer?” Biotechnology and Bioengineering 102, no. 4 (2009): 1182–1196. 18979540 10.1002/bit.22158

[11] L. M. Barnes , C. M. Bentley , and A. J. Dickson , “Molecular Definition of Predictive Indicators of Stable Protein Expression in Recombinant NS0 Myeloma Cells,” Biotechnology and Bioengineering 85, no. 2 (2004): 115–121. 14704993 10.1002/bit.10893

[12] B. M. Turner , “Defining an Epigenetic Code,” Nature Cell Biology 9, no. 1 (2007): 2–6. 17199124 10.1038/ncb0107-2

[13] J. T. Attwood , R. L. Yung , and B. C. Richardson , “DNA Methylation and the Regulation of Gene Transcription,” Cellular and Molecular Life Sciences: CMLS 59, no. 2 (2002): 241–257. 11915942 10.1007/s00018-002-8420-z PMC11146104

[14] Y. Hu , Q. An , Y. Guo , et al., “Simultaneous Profiling of mRNA Transcriptome and DNA Methylome From a Single Cell,” in Methods in Molecular Biology, ed. V. Proserpio , (New York: Springer, 2019), 363–377, 10.1007/978-1-4939-9240-9_21. 31028648

[15] R. Murr , “Interplay Between Different Epigenetic Modifications and Mechanisms,” Advances in Genetics 70 (2010): 101–141. 20920747 10.1016/B978-0-12-380866-0.60005-8

[16] V. Paredes , J. S. Park , Y. Jeong , J. Yoon , and K. Baek , “Unstable Expression of Recombinant Antibody During Long‐Term Culture of CHO Cells Is Accompanied by Histone H3 Hypoacetylation,” Biotechnology Letters 35, no. 7 (2013): 987–993. 23468139 10.1007/s10529-013-1168-8

[17] A. Osterlehner , S. Simmeth , and U. Göpfert , “Promoter Methylation and Transgene Copy Numbers Predict Unstable Protein Production in Recombinant Chinese Hamster Ovary Cell Lines,” Biotechnology and Bioengineering 108, no. 11 (2011): 2670–2681. 21618470 10.1002/bit.23216

[18] F. Saunders , B. Sweeney , M. N. Antoniou , P. Stephens , and K. Cain , “Chromatin Function Modifying Elements in an Industrial Antibody Production Platform—Comparison of UCOE, MAR, STAR and cHS4 Elements,” PLoS ONE 10, no. 4 (2015): e0120096. 25849659 10.1371/journal.pone.0120096 PMC4388700

[19] M. Antoniou , L. Harland , T. Mustoe , et al., “Transgenes Encompassing Dual‐Promoter CpG Islands from the Human TBP and HNRPA2B1 Loci Are Resistant to Heterochromatin‐Mediated Silencing,” Genomics 82, no. 3 (2003): 269–279. 12906852 10.1016/s0888-7543(03)00107-1

[20] S. Williams , T. Mustoe , T. Mulcahy , et al., “CpG‐Island Fragments from the HNRPA2B1/CBX3 Genomic Locus Reduce Silencing and Enhance Transgene Expression from the hCMV Promoter/Enhancer in Mammalian Cells,” BMC Biotechnology 5 (2005): 17. 15935093 10.1186/1472-6750-5-17 PMC1175082

[21] S. S. Rudina and C. D. Smolke , “A Novel Chromatin‐Opening Element for Stable Long‐Term Transgene Expression,” bioRxiv, May 3, (2019), 10.1101/626713.

[22] T. Benton , T. Chen , M. McEntee , et al., “The Use of UCOE Vectors in Combination with a Preadapted Serum Free, Suspension Cell Line Allows for Rapid Production of Large Quantities of Protein,” Cytotechnology 38, no. 1–3 (2002): 43–46. 19003085 10.1023/A:1021141712344 PMC3449923

[23] L. Harland , R. Crombie , S. Anson , J. deBoer , P. A. Ioannou , and M. Antoniou , “Transcriptional Regulation of the Human TATA Binding Protein Gene,” Genomics 79, no. 4 (2002): 479–482. 11944977 10.1006/geno.2002.6728

[24] D. J. Simpson , S. G. Williams , and A. S. Irvine “Expression Elements,” (USPTO Patent No. 7632661), (2009).

[25] S. Majocchi , E. Aritonovska , and N. Mermod , “Epigenetic Regulatory Elements Associate with Specific Histone Modifications to Prevent Silencing of Telomeric Genes,” Nucleic Acids Research 42, no. 1 (2014): 193–204. 24071586 10.1093/nar/gkt880 PMC3874193

[26] S. Boscolo , F. Mion , M. Licciulli , et al., “Simple Scale‐Up of Recombinant Antibody Production Using an UCOE Containing Vector,” New Biotechnology 29, no. 4 (2012): 477–484. 22226921 10.1016/j.nbt.2011.12.005

[27] K. A. Skipper , A. K. Hollensen , M. N. Antoniou , and J. G. Mikkelsen , “Sustained Transgene Expression from Sleeping Beauty DNA Transposons Containing a Core Fragment of the HNRPA2B1‐CBX3 Ubiquitous Chromatin Opening Element (UCOE),” BMC Biotechnology 19, no. 1 (2019): 75. 31706316 10.1186/s12896-019-0570-2 PMC6842454

[28] R. Hoseinpoor , B. Kazemi , M. Rajabibazl , and A. Rahimpour , “Improving the Expression of Anti‐IL‐2Rα Monoclonal Antibody in the CHO Cells through Optimization of the Expression Vector and Translation Efficiency,” Journal of Biotechnology 324 (2020): 112–120. 33007349 10.1016/j.jbiotec.2020.09.006

[29] A. R. Nair , X. Jinger , and T. W. Hermiston , “Effect of Different UCOE‐Promoter Combinations in Creation of Engineered Cell Lines for the Production of Factor VIII,” BMC Research Notes 4 (2011): 178. 21663669 10.1186/1756-0500-4-178 PMC3138458

[30] Z. Betts , A. S. Croxford , and A. J. Dickson , “Evaluating the Interaction Between UCOE and DHFR ‐Linked Amplification and Stability of Recombinant Protein Expression,” Biotechnology Progress 31, no. 4 (2015): 1014–1025. 25829363 10.1002/btpr.2083

[31] R. E. Sizer , R. M. Ingram , C. Swan , E. K. Biggs , L. P. Pybus , and R. J. White , “Use of tRNA Gene Barriers Improves Stability of Transgene Expression in CHO Cells,” Biotechnology Journal 19, no. 8 (2024): 2400196, 10.1002/biot.202400196. 39115350

[32] J. R. Raab , J. Chiu , J. Zhu , et al., “Human tRNA Genes Function as Chromatin Insulators,” EMBO Journal 31, no. 2 (2012): 330–350. 22085927 10.1038/emboj.2011.406 PMC3261562

[33] S. Ghisletti , I. Barozzi , F. Mietton , et al., “Identification and Characterization of Enhancers Controlling the Inflammatory Gene Expression Program in Macrophages,” Immunity 32, no. 3 (2010): 317–328. 20206554 10.1016/j.immuni.2010.02.008

[34] J. M. Simon , P. G. Giresi , I. J. Davis , and J. D. Lieb , “Using Formaldehyde‐Assisted Isolation of Regulatory Elements (FAIRE) to Isolate Active Regulatory DNA,” Nature Protocols 7, no. 2 (2012): 256–267. 22262007 10.1038/nprot.2011.444 PMC3784247

[35] A. R. Mansisidor and V. I. Risca , “Chromatin Accessibility: Methods, Mechanisms, and Biological Insights,” Nucleus 13 (2022): 236–276. 36404679 10.1080/19491034.2022.2143106 PMC9683059

[36] J. S. Becker , D. Nicetto , and K. S. Zaret , “H3K9me3‐Dependent Heterochromatin: Barrier to Cell Fate Changes,” Trends in Genetics: TIG 32, no. 1 (2016): 29–41. 26675384 10.1016/j.tig.2015.11.001 PMC4698194

[37] Y. Cai , Y. Zhang , Y. P. Loh , et al., “H3K27me3‐Rich Genomic Regions Can Function as Silencers to Repress Gene Expression via Chromatin Interactions,” Nature Communications 12, no. 1 (2021): 1–22. 10.1038/s41467-021-20940-y PMC7846766 33514712

[38] I. B. Hilton , A. M. D'Ippolito , C. M. Vockley , et al., “Epigenome Editing by a CRISPR‐Cas9‐Based Acetyltransferase Activates Genes from Promoters and Enhancers,” Nature Biotechnology 33, no. 5 (2015): 510–517. 10.1038/nbt.3199 PMC4430400 25849900

[39] K. Wang , M. Escobar , J. Li , et al., “Systematic Comparison of CRISPR‐Based Transcriptional Activators Uncovers Gene‐Regulatory Features of Enhancer–Promoter Interactions,” Nucleic Acids Research 50, no. 14 (2022): 7842–7855. 35849129 10.1093/nar/gkac582 PMC9371918

[40] H. O'Geen , M. Tomkova , J. A. Combs , E. K. Tilley , and D. J. Segal , “Determinants of Heritable Gene Silencing for KRAB‐dCas9 + DNMT3 and Ezh2‐dCas9 + DNMT3 Hit‐and‐run Epigenome Editing,” Nucleic Acids Research 50, no. 6 (2022): 3239–3253. 35234927 10.1093/nar/gkac123 PMC8989539

[41] J. Walther , J. Lu , M. Hollenbach , et al., “Perfusion Cell Culture Decreases Process and Product Heterogeneity in a Head‐to‐Head Comparison with Fed‐Batch,” Biotechnology Journal 14, no. 2 (2019): e1700733. 29851298 10.1002/biot.201700733

[42] J. S. Donaldson , M. P. Dale , and S. J. Rosser , “Decoupling Growth and Protein Production in CHO Cells: A Targeted Approach,” Frontiers in Bioengineering and Biotechnology 9 (2021): 658325. 34150726 10.3389/fbioe.2021.658325 PMC8207133

[43] S. Williams , T. Mustoe , T. Mulcahy , et al., “CpG‐Island Fragments from the HNRPA2B1/CBX3 Genomic Locus Reduce Silencing and Enhance Transgene Expression from the hCMV Promoter/Enhancer in Mammalian Cells,” BMC Biotechnology 5, no. 1 (2005): 17. 15935093 10.1186/1472-6750-5-17 PMC1175082

[44] Z. Betts and A. J. Dickson , “Assessment of UCOE on Recombinant EPO Production and Expression Stability in Amplified Chinese Hamster Ovary Cells,” Molecular Biotechnology 57, no. 9 (2015): 846–858. 26088164 10.1007/s12033-015-9877-y

[45] U. Müller‐Kuller , M. Ackermann , S. Kolodziej , et al., “A Minimal Ubiquitous Chromatin Opening Element (UCOE) Effectively Prevents Silencing of Juxtaposed Heterologous Promoters by Epigenetic Remodeling in Multipotent and Pluripotent Stem Cells,” Nucleic Acids Research 43, no. 3 (2015): 1577–1592. 25605798 10.1093/nar/gkv019 PMC4330381

[46] Z. Betts and A. J. Dickson , “Ubiquitous Chromatin Opening Elements (UCOEs) Effect on Transgene Position and Expression Stability in CHO Cells Following Methotrexate (MTX) Amplification,” Biotechnology Journal 11, no. 4 (2016): 554–564. 26632501 10.1002/biot.201500159

[47] J. L. Miller and P. A. Grant , “The Role of DNA Methylation and Histone Modifications in Transcriptional Regulation in Humans,” Sub‐Cellular Biochemistry 61 (2013): 289–317. 23150256 10.1007/978-94-007-4525-4_13 PMC6611551

[48] C. Mertens and R. G. Roeder , “Different Functional Modes of p300 in Activation of RNA Polymerase III Transcription from Chromatin Templates,” Molecular and Cellular Biology 28, no. 18 (2008): 5764–5776. 18644873 10.1128/MCB.01262-07 PMC2546916

[49] R. E. Sizer and R. J. White , “Use of Ubiquitous Chromatin Opening Elements (UCOE) as Tools to Maintain Transgene Expression in Biotechnology,” Computational and Structural Biotechnology Journal 21 (2022): 275–283. 36582439 10.1016/j.csbj.2022.11.059 PMC9764128

[50] N. Gödecke , S. Herrmann , H. Hauser , A. Mayer‐Bartschmid , M. Trautwein , and D. Wirth , “Rational Design of Single Copy Expression Cassettes in Defined Chromosomal Sites Overcomes Intraclonal Cell‐to‐Cell Expression Heterogeneity and Ensures Robust Antibody Production,” ACS Synthetic Biology 10, no. 1 (2021): 145–157. 33382574 10.1021/acssynbio.0c00519

[51] F. Nematpour , F. Mahboudi , B. Vaziri , et al., “Evaluating the Expression Profile and Stability of Different UCOE Containing Vector Combinations in mAb‐producing CHO Cells,” BMC Biotechnology 17, no. 1 (2017): 18. 28228095 10.1186/s12896-017-0330-0 PMC5322649

