# Supplementary material for: Barriers Composed of tRNA Genes Can Complement the Benefits of a Ubiquitous Chromatin Opening Element to Enhance Transgene Expression
Source: Biotechnol J. 2025 Feb 16;20(2):e202400455. doi: 10.1002/biot.202400455 (PMC11830863; doi:10.1002/biot.202400455)
Supplement: Supplementary file 1 — Supporting Information [file BIOT-20-e202400455-s001.pdf]

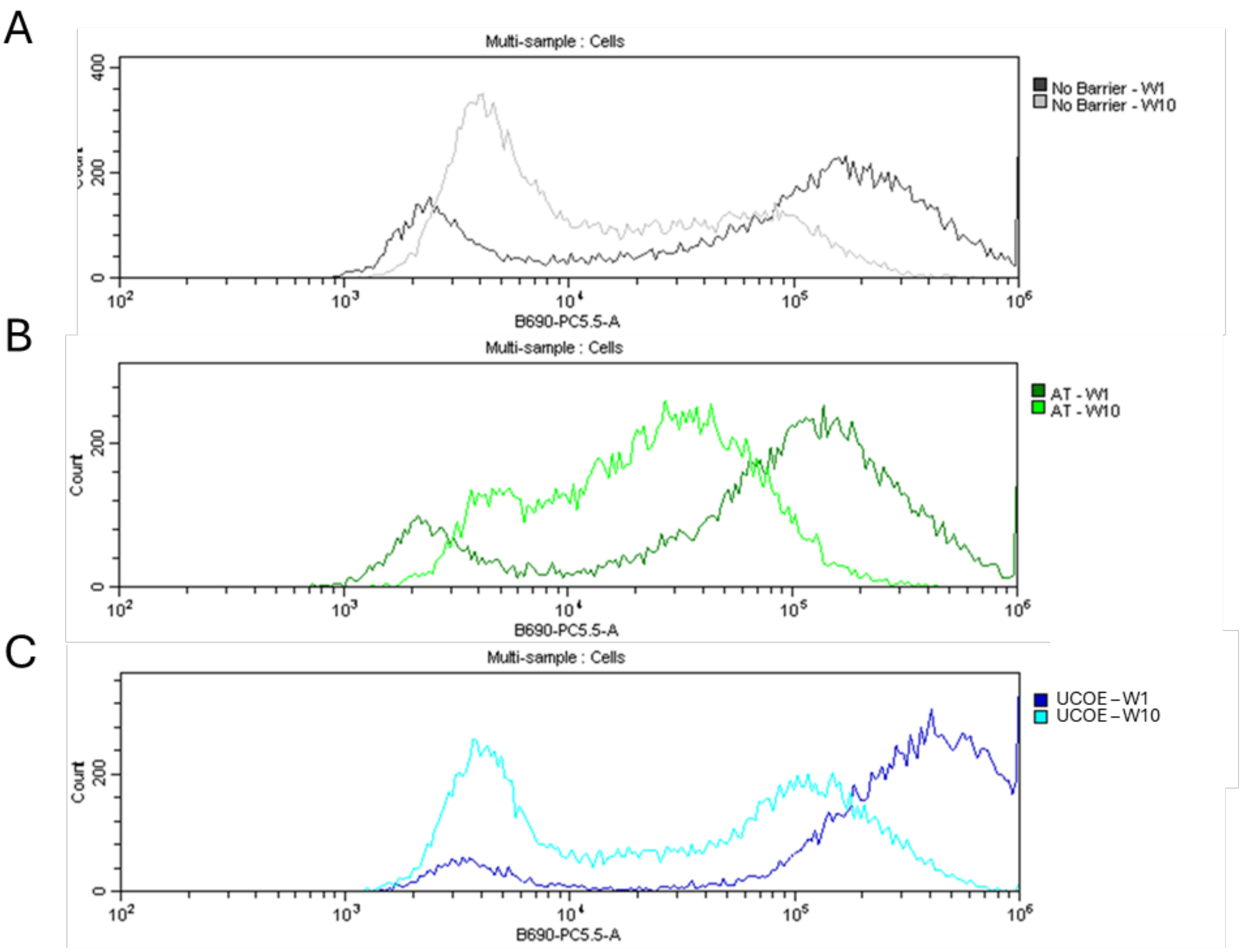

FIGURE S1: Barrier elements promote expression of an eGFP reporter gene. (A-C) Histograms showing eGFP fluorescence one week (W1) and ten weeks (W10) after selection is removed from cells transfected with eGFP transgenes with no barrier (A), flanked by AT tRNA genes (B), or downstream of a UCOE (C).
